# Dual contraception method utilization and associated factors among sexually active women on antiretroviral therapy in Gondar City, northwest, Ethiopia: a cross sectional study

**DOI:** 10.1186/s12905-020-0890-3

**Published:** 2020-02-12

**Authors:** Fewuze Abay, Hedija Yenus Yeshita, Fantahun Ayenew Mekonnen, Mekonnen Sisay

**Affiliations:** 1 Department of Reproductive Health, Institute of Public Health, College of Medicine and Health Sciences, University of Gondar, Gondar, Ethiopia; 2 Department of Epidemiology and Biostatistics, Institute of Public Health, College of Medicine and Health Sciences, University of Gondar, Gondar, Ethiopia; 3 Department of Human Nutrition, Institute of Public Health, College of Medicine and Health Sciences, University of Gondar, Gondar, Ethiopia

**Keywords:** Dual contraceptive utilization, Women on antiretroviral therapy, Gondar, Ethiopia

## Abstract

**Background:**

Mother to child transmission is responsible for 90% of child infection with human immune deficiency virus (HIV). Dual contraceptive use is one of the best actions to prevent mother’s human immune deficiency virus transmission to her child and partner. This study aimed at assessing the prevalence and factors associated with dual contraceptive use among sexually active women on antiretroviral therapy in Gondar City, northwest, Ethiopia.

**Methods:**

An institution based cross sectional study was conducted in Gondar City public health facilities from December 1 to 31, 2018. Systematic random sampling technique was utilized to include 563 study participants. Data were collected by interview using a structured questionnaire. Descriptive analysis was made to compute mean, median and proportion. Finally, multivariable logistic regression model was fitted to identify the factors associated with dual contraceptive method utilization. Analysis was performed by using Statistical Package for Social Sciences (SPSS) software version 20.

**Results:**

The overall prevalence of dual contraceptive method utilization among sexually active women on antiretroviral therapy was 28.8% (95% CI: 24.9, 32.7). Women aged 35–49 years (Adjusted odds ratio (AOR): 6.99; 95% CI: 3.11, 15.71)), who lived in urban areas (AOR: 4.81; 95% CI: 2.04, 11.31), attended secondary and above education (AOR: 4.43; 95% CI: 1.92, 10.22), and disclosed HIV status to sexual partners (AOR: 9.84; 95% CI: 3.48, 27.81) were more likely to use dual contraceptive method.

**Conclusion:**

In this study, the proportion of women who utilized dual contraceptive method was low. Age, place of residence, educational status and disclosure of HIV status were factors associated with dual contraceptive use. Therefore, providing education about the advantages of disclosing HIV status to sexual partners and strengthening of counseling about the advantages of dual contraceptive use will be helpful in enhancing the use of dual contraceptive method among sexually active women on antiretroviral therapy.

## Background

According to 2015 Global statistics, 36.7 million people were living with human immune deficiency virus (HIV) worldwide [1], of whom, reproductive age group contributed 75% of the burden [2]. The problem is highest among people residing in Sub Saharan Africa where, 60% of people reported to live with human immune deficiency virus/acquired immune deficiency syndrome (HIV/AIDS) and more than half of them were females [3]. In Ethiopia, the total number of people living with HIV was documented to be 769, 600 in 2014. Of these, about 60% were females [4].

HIV/AIDS continues to have disastrous medical, economic, social, and physical impacts on individuals, females, nations and global community at large [5]. Globally, more than 2 million HIV positive women become pregnant every year due to poor contraceptive utilization and unsafe sex practices, out of which, 600, 000 die due to pregnancy related complications [6]. Unintended pregnancies accounted for 21.3% of new paediatric HIV infections [7], 90% were from Sub Saharan Africa [8]. Ethiopia is one of the countries severely affected by the disease with over 100, 000 pregnancies tested positive for HIV and over 12, 000 HIV positive births annually [9]. Unintended pregnancies among HIV infected women contribute to poor maternal and child outcomes [10, 11].

In Sub Saharan Africa increasing the contraceptive prevalence rate (CPR) has been estimated to reduce the proportion of infants infected with HIV by 35–55% through reduction in primary HIV infection and unintended pregnancies in HIV infected women [12]. This reduction rate can be possible through exercising consistent use of male condom alone which can protect both unwanted pregnancies and sexually transmitted infections (STIs)/HIV at the same time [13]. However, using it alone as a contraceptive method is not a guarantee to be protected from unwanted pregnancy since inconsistent and improper use is the common practice [14]. For example, about 15% of a one-year cumulative incidence of unintended pregnancy had occurred as a result of using it alone [15]. To add more, among those who had routine use of condom alone, about 14 to 21% of them got pregnant during the first year [14, 16].

Even though, HIV-positive women can use other hormonal and non-hormonal contraceptive methods rather than male condom for preventing pregnancy, these methods do not protect partners against the transmission of both STIs/HIV between them [15, 17]. Consequently, it is true that there is no a single type of contraceptive method which is effective at preventing both unintended pregnancies and STIs/HIV at a time. Thus, dual-contraceptive method use is the most effective approach and strategy to effectively prevent both unwanted pregnancies and STIs/HIV simultaneously [18, 19].

Dual contraceptive method use is the use of two different types of contraceptive methods, a barrier contraceptive along with another family planning method which can reduce transmission of STIs/HIV and prevent pregnancy respectively [20, 21]. Evidences show that 40 and 80% of unplanned pregnancies and abortions would be prevented if half and all of them who were using one type of contraceptive alone started using dual methods [22]. An intervention based study had also demonstrated that participants who adhered to dual method use had lower rates of unintended pregnancy at 24 months [23].

Apart from its effectiveness, its utilization is low particularly in developing countries including Ethiopia [24–28]. Several recent studies conducted in different parts of Ethiopia have examined dual contraceptive method use among HIV positive women and reported low utilization. A study done at the University of Gondar hospital, northwest Ethiopia, a study conducted in selected public hospitals of northern Ethiopia and another study done in Gebretsadik Shawo hospital, southwest Ethiopia found out that about 13.2, 15.7 and 19.8% of HIV positive women who were attending antiretroviral therapy (ART) clinics used dual contraceptive method, respectively [4, 29, 30].

Previous studies have reported that unmarried women [31], women who disclosed their HIV status to their partners, sero-discordant and no desire for more children [32–34] were more likely to use dual contraceptive method.

Currently, in Ethiopia, HIV transmission is high and is a public health problem. Many HIV positive women are still encountering unintended pregnancy with a concomitant risk of mother to child transmission (MTCT) of HIV, pregnancy related morbidity and mortality, and transmission of HIV and new strain of HIV virus to their sexual partners due to low dual contraceptive method utilization. Ethiopian national health policy and strategy encourage dual contraceptive method use as a key intervention to reduce HIV transmission and unintended pregnancy. In addition, Ministry of health of Ethiopia in collaboration with partners also developed the post 2015 objective of prevention of mother to child transmission (PMTCT) to consolidate & sustain the elimination of MTCT and reduce the vertical transmission to less than 2% by 2020. To ensure virtual elimination, one of the main focuses is improving the use of dual contraceptive method among HIV positive women by integrating family planning services with PMTCT [35]. However, there is no adequate evidence in Ethiopia, particularly in our study area, Gondar City.

Therefore, the results of the current work would be helpful by providing updated evidences regarding the prevalence of dual contraceptive method utilization and its associated factors which hinder and/or enhance its utilization among sexually active women on antiretroviral therapy (ART), in Gondar City, northwest Ethiopia to public health practitioners and policy makers to create programs and restructure the system to reach the target audiences.

## Methods

### Study design and setting

An institutional based cross sectional study was conducted from December 1 to 31, 2018 in Gondar City public health facilities with ART clinics. Gondar town is located in the Amhara regional State, Ethiopia at a distance of 747 km from Addis Ababa, the capital of Ethiopia. The City has a total population of 333, 103. About 2, 919 and 2, 152 reproductive age women were enrolled in ART clinics of Gondar referral hospital and Gondar City health centers, respectively [36, 37].

### Study population

All randomly selected sexually active HIV positive women of reproductive age (15–49), who had follow up in the ART clinics of Gondar City health facilities were included in this study. Women who were pregnant, had hearing difficulties and/or with confirmed infecundity were excluded.

### Sample size determination and sampling procedure

The sample size was determined by using single population proportion formula taking the assumptions of 32% dual contraceptive proportion (P) (28), 95% confidence interval (CI), 4% margin of error (d) and a 10% nonresponse rate using Epi-Info software version 7 which yielded 574 participants.

The estimated sample size was allocated to each health facility proportion to size of ART user women in the respective health facility. During the data collection period, there were a total of 1148 sexually active HIV positive women, of whom, 655 were from Gondar referral hospital and 295, 114, 47 and 37 were from Gondar, Azezo, Maraki and Teda health centers, respectively. Accordingly, 327 (655*574/1148), 148 (295*574/1148), 57 (114*574/1148), 23 (47*574/1148) and 19 (37*574/1148) samples were proportionally taken from Gondar referral hospital, Gondar, Azezo, Maraki and Teda health centers, respectively. A systematic random sampling technique was used to select participants from each health facilities. Sampling interval (K) was determined by dividing the total number of pregnant women who were visiting the ART clinics by the estimated sample size; K = 1148/574 = 2. The first participant was identified by lottery method from 1 and 2, and 2 was drawn as the first participant. Then, every 2 participants were selected from the sampling frame until the total sample size was achieved.

### Data collection procedures and quality control

A structured questionnaire was used to collect the data (Additional file 1). It was composed of socio demographic and economic factors (age, marital status, residence, religion, woman’s educational level, partner’s educational level, woman’s occupation, partner’s occupation and income), sexual and reproductive factors (number of children, woman’s desire to have children, partner’s desire to have children and knowledge about contraceptive), and factors related to HIV and STI (CD4 cell count, duration of ART, viral load and history of STI, knowledge of partner’s HIV status, HIV status disclosure to partner). The questionnaire was originally prepared in English and translated to Amharic and back to English to ensure consistency. Five diploma and two-degree holder nurses were recruited as data collectors and supervisors, respectively. The data were collected after a one-day training was given regarding interview techniques and ethical concerns. Chart review was done for medical records of participants, including confirmation of highly active antiretroviral therapy (HAART), duration of ART, STI history, CD4cell and viral load counts. The questionnaire was pretested on 5% of the total sample from similar participants outside of Gondar City. Then, necessary modifications were done to the questionnaire. The data collection process was closely supervised by the supervisors and principal investigator on a daily basis.

### Measurement of variables and operational definitions

#### Dual contraceptive method use

Utilization of any hormonal, intrauterine contraceptive device or permanent modern contraceptive method along with male or female condom for the last 1 month prior to the study period [38].

#### Knowledge of contraceptive

Respondents, who have heard of at least one contraceptive method [4, 39].

#### Sexually active

A client who engaged in sexual activity within 1 month prior to the survey [40].

### Data processing and analysis

Data were coded, cleaned, and entered in to Epi-Info version 7, and then exported to SPSS software version 20 for analysis. Summary statistics of variables such as percentage, median and Inter Quartile Range (IQR) were calculated. Multivariable analysis was used to identify associated factors of dual contraceptive utilization. Effect size was presented by odds ratio (OR) with 95% confidence interval and *p*-value. A predictor variable with *p*-value less than 0.05 was considered as having a statistically significant association with the dependent variable. Furthermore, model fitness was checked by Hosmer and Lemeshow goodness of fit-test (*p* = 0.35).

### Ethical considerations

Ethical clearance was obtained from the Institutional Review Board of the University of Gondar. Permission letter was obtained from Gondar City Administration Health Office. Each health facility head was communicated through formal letter obtained from Gondar City Administration Health Office. Objectives of the study were explained to study participants. Written informed consent was obtained from participants, or parents/guardians of participants for those whose ages were less than 18 years old. The confidentiality of information was guaranteed by using code numbers rather than personal identifiers and by keeping the data protected by locking. Participants were told that they could decline at any time if they feel uncomfortable, even after the interview has started.

## Results

### Socio, demographic characteristics of respondents

A total of 563 women on ART participated in this study with a response rate of 98%. The remaining participants provided incomplete responses and were rejected from the analysis. The median age of the respondents was 31 (IQR; 26, 37) years. Half of the participants were found in the age category of 25–34 years. Below one fifth, 97 (17.2%) and 184 (32.7%) of the participants were in the age category of 35 and above and 15–24 years, respectively. About half, 282 (50.1%) of them were also between 25 and 34 years. Regarding to their monthly income, 185 (32.9%), 207 (36.8%), 162 (28.8%), and 9 (1.6%) had > 2500, 1501–2500, 501–1500, and < =500 Ethiopian Birr, respectively.

Four hundred seventy-six (84.5%) women were urban residents. The majority, 514 (91.3%) and 546 (97%), of the participants belong to Orthodox Christian religion and Amhara ethnic, respectively. Four hundred eighty-six (86%) women were married (Table 1).

**Table 1:** Socio- demographic characteristics of sexually active women on ART in public health facilities of Gondar City, northwest, Ethiopia, 2018 (*n* = 563)

Variables	Frequency (n)	Percent (%)
Age		
15–24	97	17.2
25–34	282	50.1
> =35	184	32.7
Residence		
Urban	476	84.5
Rural	87	15.5
Religion		
Orthodox	514	91.3
Muslim	49	8.7
Marital status		
Married	486	86.3
Never married	51	9.1
Divorced	17	3.0
Separated	9	1.6
Ethnicity		
Amhara	546	97.0
Tigray	10	1.8
Other^a^	7	1.2
Women’s education		
No education	245	43.5
Primary education	183	32.5
Secondary education	94	16.7
Above secondary	41	7.3
Husband’s education		
No education	226	40.1
Primary education	212	37.7
Secondary education	107	19.0
Above secondary	18	3.2
Woman’s occupation		
Student	16	2.8
Merchant	100	17.8
Government employee	49	8.7
Housewife	184	32.7
Daily laborer	82	14.6
Private employee	102	18.1
Other^b^	30	5.3
Husband’s occupation		
Student	3	0.5
Merchant	122	21.7
Government employee	78	13.9
Daily laborer	144	25.6
Private employee	185	32.9
Other^c^	31	5.5
Income		
< =500	9	1.6
501, 1500	162	28.8
1501, 2500	207	36.8
> 2500	185	32.9

Note: Other ^a^Oromo, ^b^Farmer, No occupation, ^c^Waitress, Farmer

### Sexual and reproductive characteristics

Regarding women’s knowledge about contraceptive method, all, 563 (100%) of study participants knew at least one method of contraception. The majority, 465 (82.6%) of study participants and 359 (63.8%) of women’s sexual partners had no desire to have more children in the future.

### Clinical and HIV related factors

Concerning their sexual partners’ HIV status, more than two third, 403 (71.6%) of them reported to aware their partners’ HIV status. The majority, 393 (69.8%) of women disclosed their HIV status to their sexual partners. Of all participants, only 34 (6%) of them had history or symptom of other STIs in the past 1 year, 30 (88.2%) of whom received treatment (Table 2).

**Table 2:** Clinical and HIV related characteristics of sexually active women on ART in public health facilities of Gondar City, northwest Ethiopia, 2018

Variables	Frequency (n)	Percent (%)
STI history		
No	529	94.0
Yes	34	6.0
Know HIV status of partner		
No	160	28.4
Yes	403	71.6
Partner’s HIV status		
Negative	164	40.7
Positive	239	59.3
Disclosed HIV status		
No	170	30.2
Yes	393	69.8
Duration on ART (Years)		
0, 1	44	7.8
2, 3	134	23.8
4, 5	75	13.3
6, 7	142	25.2
8, 9	111	19.7
> =10	57	10.1
Current CD4 cell count		
< 250	97	17.2
250, 349	109	19.4
> =350	357	63.4
Current viral load		
Not high viral load	526	93.4
High viral load	37	6.6

### Dual contraceptive method utilization

This study showed that 162 (28.8%) of the study participants (sexually active HIV positive women), used dual contraceptive method in the past 1 month prior to the survey. All, 563 (100%) of the study participants reported using at least one modern contraceptive method. Of them, 386 (68.6%) used condom and 200 (35.5%) used dual contraceptive method. Twenty-five (4.4%) used condom only. The most common methods used along with condom were, injectable 112 (69%), pills 25 (15%), implants 22 (14%) and Intra Uterine Contraceptive Device (IUCD) 3 (2%).

### Factors associated with dual contraceptive use

Both bivariable and multivariable binary logistic regression models were used to conduct the crude and adjusted odds ratios with their 95% confidence intervals (CI) and *p*-values. Accordingly, the result of bivariable analysis showed that age, residence, and educational status of the woman, occupation of partner, knowledge of partner’s HIV status, HIV status of the partner and disclosure of HIV status to sexual partner were associated with dual contraceptive utilization. However, in multivariable logistic regression analysis, age, residence, educational status of the woman, and disclosure of HIV status to sexual partner were statistically significantly associated with dual contraceptive utilization.

Consequently, women in the age group of 35–49 years were 7 times more likely to use dual contraceptive method than those in the age group of 15 to 24 years (AOR: 6.99; 95% CI: 3.11, 15.71). Similarly, women who were urban residents were nearly 5 times more likely to use dual contraceptive method compared to their rural resident counterparts (AOR: 4.80; 95% CI: 2.04, 11.31). Likewise, higher odds of dual contraceptive method utilization were observed among women who attended secondary and above education than those who attended lower education (AOR: 4.43; 95% CI: 1.92, 10.22). Again, the odds of dual contraceptive method utilization were nearly 10 times higher among those who disclosed their HIV status to their sexual partners (AOR: 9.84; 95% CI: 3.48, 27.81) (Table 3).

**Table 3:** Factors associated with dual contraceptive use among sexually active women on ART in public health facilities of Gondar City, northwest Ethiopia, 2018

Variable	Dual contraceptive use (*n* = 563)		Crude OR (95% CI)	Adjusted OR (95% CI)	*P*-value
	Yes (%)	No (%)			
Age (years)					
15, 24	11 (11.3)	86 (88.7)	1	1	
25, 34	65 (23)	217 (77)	2.34 (1.18, 4.65)*	2.11 (0.97, 4.55)	0.058
35, 49	86 (46.7)	98 (53.3)	6.86 (3.44, 13.71)*	6.99 (3.11, 15.71)**	< 0.001
Residence					
Urban	154 (32.4)	322 (67.6)	4.72 (2.23, 10.02)*	4.8 (2.04, 11.31)**	< 0.001
Rural	8 (9.2)	79 (90.8)	1	1	
Educational status of woman					
No formal education	51 (20.8)	194 (79.2)	1	1	
Primary education	44 (24)	139 (76)	1.2 (0.76, 1.91)	1.03 (0.54, 1.98)	0.921
Secondary and above	46 (48.9)	48 (51.1)	3.65 (2.19, 6.06)*	4.43 (1.92, 10.22)**	< 0.001
Partner’s occupation					
Student	1 (33.3)	2 (66.7)	0.53 (0.04, 6.51)	2.5 (0.14, 46.11)	0.537
Merchant	31 (25.4)	91 (74.6)	0.36 (0.16, 0.82)*	0.72 (0.26, 1.98)	0.53
Government employee	21 (26.9)	57 (73.1)	0.39 (0.17, 0.93)*	0.59 (0.20, 1.73)	0.337
Daily labor	41 (28.5)	103 (71.5)	0.43 (0.19, 0.94)*	1.05 (0.37, 2.88)	0.918
Private employee	53 (28.6)	132 (71.4)	0.43 (0.21, 0.93)*	0.63 (0.24, 1.65)	0.348
Other*	15 (48.4)	16 (51.6)	1	1	
Aware of partner’s HIV status					
No	7 (4.4)	153 (95.6)	1	1	
Yes	155 (38.5)	248 (61.5)	13.66 (6.24, 29.91)*	2.69 (1.01, 7.31)	0.051
Disclosure of HIV status					
No	6 (3.5)	164 (96.5)	1	1	
Yes	156 (39.7)	237 (60.3)	17.99 (7.77, 41.65)*	9.84 (3.48, 27.81)**	< 0.001

Note: *Crude OR, Significant at *P* value < 0.05, **Adjusted OR, Significant at *P* value < 0.05

## Discussion

This study was conducted to assess the proportion of dual contraceptive utilization and its associated factors among sexually active women on ART in Gondar City health facilities, northwest Ethiopia. The study revealed that, 28.8% (95% CI: 24.9–32.7) of sexually active women on ART used dual contraceptive method in the last month prior to the study. The factors identified to be significantly associated with dual contraceptive use were women’s age, residence, educational status and disclosure of HIV status to sexual partner.

The proportion of dual contraceptive use in this study is consistent with the results of studies done in Fitche (32%) and Addis Ababa (31%), Ethiopia, and in Nigeria (27.2%), and India (30%) [10, 34, 41, 42]. But, the percentage of women using dual method is higher in our study compared to the findings of studies conducted in Tigray region (14%), Mekelle public hospitals (15.7%), and Gimbie town (17%), Ethiopia and in Zambia (17.7%) [4, 39, 40, 43]. The observed variation between the studies might be due to the differences in socio demographic characteristics of participants included. Again, the inconsistency noted with studies from other countries may be attributable to socio demographic and cultural characteristics of study participants, study period and settings.

The proportion of women using dual method is lower in the present study compared to the findings of studies done in Tigray (59.9%), Ethiopia, Kenya (38.5%), and Nigeria (45%) [32, 44, 45].. The discrepancy between study reports could probably be due to socio-demographic and cultural differences of the study populations, presence of quality, integrated sexual and reproductive health and ART services in Kenya and Nigeria.

According to our study results, older age women (35 to 49 years) were more likely to use dual contraceptive method than younger age women (15 to 24 years). The possible reason could be older age women might have better understanding the advantage of using dual contraceptives, in turn, making them use the method. In addition, there is an established evidence that as age advances the desire for more children in the future decreases. This finding suggested that providing due attention for younger age women is crucial to improve the utilization of dual contraceptive method among HIV positive women. This finding is in line with studies reported from southern part of Ethiopia, Uganda and Brazil [30, 33, 46].

Place of residence was one of the key determinants of dual contraceptive method utilization. This can be explained by the fact that urban residents are more exposed for better health information and technologies which might enable them to have a better decision power and free discussion with their sexual partners about utilization of family planning services than their rural counterparts. Furthermore, women living in urban areas are believed to have better physical access to contraceptives. Such explanation suggested the importance of enhancing counselling services focussing on rural women to increase the uptake of dual contraceptive method utilization. This evidence is supported by a study done in southern part of Ethiopia [30] which revealed that HIV positive urban women were more likely to use dual contraceptive method than rural women.

Higher proportion of dual contraceptive method utilization was observed among women who attended secondary and above education. This can be explained as women with high level of education might appreciate the advantages of dual contraceptive use, have higher levels of HIV/AIDS knowledge, able to discuss freely with their sexual partners and are less likely to have stigma towards HIV/AIDS, which, in turn, assists them to easily change their risky sexual behaviour. This result implied that the paramount contribution of women’s education to improve dual contraceptive method utilization. Similar results were reported from Brazil and Uganda [33, 46].

This study also showed that disclosing own HIV status to sexual partner has been found to significantly increase utilization of dual contraceptive method. This may be due to the fact that women have common understanding about the importance of using dual contraceptive method and easily reach at consensus to use it. Moreover, disclosing HIV status fosters more open discussion with their sexual partners with regard to safe sexual practice and limiting number of children. As the study showed, disclosing their HIV status to their respective sexual partners is crucial to increase the coverage of dual contraceptive method among HIV positive women. This finding is supported by studies conducted in Mekelle and Addis Ababa, Ethiopia, Ghana, and Kenya [32, 40, 47, 48].

## Conclusion

The proportion of sexually active HIV positive women who were using dual contraceptive method was low. Women’s age, educational status, place of residence, and disclosure of HIV status to sexual partners were found to be significantly associated with dual contraceptive method utilization. Strengthening education of women on ART about the importance of disclosing own HIV status to sexual partner in particular and the advantage of using dual contraceptive in general is suggested for the prevention of the transmission of HIV to the child and sexual partner. Furthermore, encouraging and providing more attention for younger age, illiterate and rural women by health care providers and other concerned bodies is recommended.

### Limitations of the study

This study tried to generate evidences on dual contraceptive utilization among HIV positive women. However, it cannot be free from some limitations. Due to the cross-sectional nature of the study design, temporal relationship could not be assessed. Since the issue is a sensitive one, this result may be prone to social desirability bias. In addition, variables such as social support awareness towards pregnancy and STDs, financial support, availability of services/supplies and involvement of association of people living with HIV/AIDS that might have been associated with dual contraceptive use were not included. Furthermore, the current study was health facility based, the result of this study may not be generalizable to those HIV positive mothers who were attending health institutions outside the study area and those who were in the community.

## Supplementary information

**Additional file 1.** Questionnaire (English Version Questionnaire).

## Data Availability

Data will be available upon request from the corresponding author.

## Abbreviations

AIDS: Acquired immune deficiency syndrome
AOR: Adjusted Odds Ratio
ART: Antiretroviral therapy
CI: Confidence Interval
COR: Crude Odds Ratio
CPR: Contraceptive Prevalence Rate
HAART: Highly active antiretroviral therapy
HIV: Human immune deficiency virus
IQR: Inter Quartile Range
IUCD: Intra uterine contraceptive device
MTCT: Mother to child transmission
PMTCT: Prevention of mother to child transmission
SPSS: Statistical Package for Social Sciences
STI: Sexually transmitted infection
